# Differential regulation of the sphere formation and maintenance of cancer-initiating cells of malignant mesothelioma via CD44 and ALK4 signaling pathways

**DOI:** 10.1038/s41388-018-0405-y

**Published:** 2018-07-30

**Authors:** Yoshiya Ohno, Serina Shingyoku, Sakina Miyake, Aya Tanaka, Sena Fudesaka, Yuta Shimizu, Ai Yoshifuji, Yuki Yamawaki, Sachiyo Yoshida, Saya Tanaka, Kazuma Sakura, Toshiyuki Tanaka

**Affiliations:** 1 Laboratory of Immunobiology, School of Pharmacy, Hyogo University of Health Sciences, Kobe, Hyogo Japan; 2 The Center of Medical Innovation and Translational Research, Osaka University Hospital, Osaka, Japan; 3 Department of Surgery, Graduate School of Medicine, Osaka University, Osaka, Japan

## Abstract

Malignant mesothelioma (MM) has a poor prognosis and is largely resistant to standard treatments, so it is important to seek novel therapeutic strategies for this disease. Cancer-initiating cells (CICs) were previously identified in MM using stem cell-associated markers in combination with spheroid cultures. However, the mechanisms underlying the induction and maintenance of CICs in MM remain to be fully explored. Here we showed that the CICs, which had high aldehyde dehydrogenase levels (ALDH^bright^) and stem cell-associated genes, were expanded in MM cells cultured under sphere-forming conditions. The MM spheroids also initiated tumors in immunodeficient mice more efficiently than did conventional adherent MM cells. In the MM spheroids, the expression of hyaluronan (HA) synthases was upregulated. Inhibiting the HA synthesis or CD44 functions by gene knockdown or neutralizing antibody abolished the formation of large-sized spheroids and the expansion of ALDH^bright^ CICs. The expression of activin-A was also increased in the spheroids, and type I activin-A receptor subunit (ALK4) was upregulated in the ALDH^bright^ CICs. The neutralization of activin-A or functional inactivation of ALK4 diminished the ALDH^bright^ CICs without affecting spheroid formation. The knockdown of CD44 or ALK4 strongly suppressed the tumor growth in immunodeficient mice. These results together suggest that the HA–CD44 and activin-A–ALK4 pathways differentially regulate the spheroid formation and maintenance of ALDH^bright^ CICs in MM cells, and that both pathways play critical roles in tumor growth in immunodeficient hosts. Our findings provide a novel therapeutic option for MM that targets signaling pathways that promote the CIC compartment through CD44 and ALK4.

## Introduction

Malignant mesothelioma (MM) is an aggressive tumor that arises primarily from the pleura, peritoneum, pericardium, or tunica vaginalis testis. Up to 80% of MM cases are of pleural origin, and are defined as malignant pleural mesotheliomas [1]. Histologically, MM is divided into three major subtypes: epithelioid, sarcomatoid, and biphasic with both epithelioid and sarcomatoid components. MM develops stealthily in patients, and is clinically diagnosed at an advanced stage of the disease after a long latency period. Because MM is largely unresponsive to standard treatments, including front-line chemotherapy with cisplatin plus pemetrexed, surgery, and radiation, the prognosis is very poor [2]. Thus, it is important to look for novel therapeutic strategies for this disease [1–3].

Overwhelming evidence indicates that asbestos exposure is the main causative agent for MM [4]. Asbestos induces several key genetic alterations in tumor suppressor genes, including CDKN2A, BAP1, and NF2, in MM cells [2]. Integrated genetic analyses showed that certain signaling pathways, such as the Hippo, mTOR, histone methylation, RNA helicase, and p53 pathways, are often affected in MM [5]. A chronic inflammatory response to asbestos also contributes to the unique tumor microenvironment of MM, which consists of tumor-surrounding extracellular matrix and secreted inflammatory cytokines [3]. Hyaluronan (HA), a widely distributed glycosaminoglycan in the extracellular matrix, is produced by MM cells and increases their malignant properties [6–8]. Among the inflammatory cytokines, activin-A, a transforming growth factor-β (TGF-β) family cytokine, has been implicated in the migration and invasive growth of MM cells [9–11].

Most cancers contain a highly tumorigenic subpopulation of cells that drive the persistence of malignant tumors by producing new cancer cells [12]. These cells, known as “cancer-initiating cells (CICs),” often acquire resistance against chemotherapeutic agents, oxidative stress, and radiation. Putative CICs of many types of solid tumors have been isolated using several cell-surface makers, including CD44, ESA, and CD133, and functional markers, such as aldehyde dehydrogenase (ALDH) and hoechst dye-excluding activity (side population) [12, 13]. In vitro studies showed that CICs can often grow into multicellular spheroids under low-attachment conditions [14]. In addition, the epithelial-to-mesenchymal transition (EMT) program was shown to be associated with CICs [12, 15].

Several studies have shown that CICs are present in MM and, using various stem cell-associated markers in combination with spheroid cultures, MM cell populations with CIC properties have been isolated [16–20]. While no universal cell-surface markers for the definite identification of CICs in MM or other types of cancers are currently available, increased ALDH1 activity characterizes cancer cell subpopulations with CIC properties in human MM cells [18–20]. However, the mechanisms underlying the induction and maintenance of CICs in MM remain to be fully explored.

In the present study, we investigated the roles of HA and activin-A and their specific receptors CD44 and ALK4, respectively, in CIC formation and maintenance using MM spheroids. We also assessed the potential of the HA–CD44 and activin-A–ALK4 axes as therapeutic targets for suppressing the CIC compartment in MM.

## Results

### The CIC population is expanded in MM spheroids

**Fig. 1:**
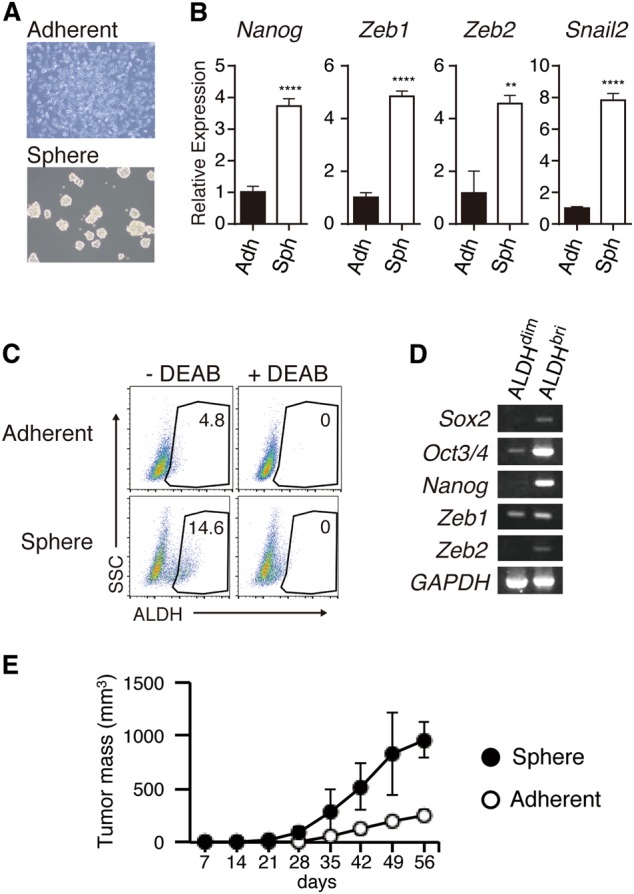
A CIC-like cell population is expanded in MSTO-211H MM spheroids. **a** MSTO-211H cells were cultured for 4 days under conventional adherent (upper) or sphere-forming conditions. **b** Total RNA was isolated from adherent cells or spheroids, and the expression of *SOX2*, *SNAIL2*, *ZEB1*, and *ZEB2* was determined by real-time PCR. Each mRNA level was normalized to that of endogenous *GAPDH*. **c** ALDH activity of adherent cells or spheroids was determined by ALDEFLUOR assay. Cell populations that disappeared with a specific ALDH inhibitor, diethylaminobenzaldehyde (DEAB), were gated as ALDH^bright^ cells. **d** Total RNA was isolated from the ALDH^bright^ or ALDH^dim^ cells sorted from spheroids, and the expression of *SNAIL2*, *ZEB1*, *SOX2*, *OCT3/4*, and *NANOG* was determined by RT-PCR. **e** Anti-CD122 (TM-β1; 500 μg/body) was intraperitoneally injected into NOD/SCID mice to deplete NK cells before tumor implantation. After 7 days, MSTO-211H cells (1 × 10^3^ cells) cultured under adherent (open circle) or spheroid (closed circle) conditions were subcutaneously injected into the mice. The tumor growth in vivo was measured every 3–4 days (*n* = 3). Data are presented in triplicate as the mean ± s.d. *P < 0.05, **P < 0.01, ***P < 0.001, ****P < 0.0001

We first examined the tumor growth of MM cell lines (ACC-MESO-1, ACC-MESO-4, NCI-H28, NCI-H2052, and MSTO-211H) in vitro and in vivo, and found that MSTO-211H cells formed tumor mass most rapidly in immunodeficient mice (Supplemental Fig. S1A and S1B). Although MESO-4 proliferated most slowly in vitro, it formed tumor mass in the mice. To investigate the cellular and molecular properties of the CIC or CIC-like cells in MM cells, we used a spheroid culture system [21]. The MM cell lines MSTO-211H, MESO-4, MESO-1, and H2052 could form spheroids, but H28 failed to do so (Fig. 1a and Supplemental Fig. S2). A comparison of MM cells in spheroid culture with those in conventional adherent culture showed that the expressions of stem cell-associated genes, including *NANOG, ZEB1, ZEB2*, and *SNAIL2*, were higher in the MM spheroids (Fig. 1b, Supplemental Fig. S2B, S2C). ALDH activity is a reliable marker for various types of cells with stem cell-like properties [13]. The ALDH^bright^ cell population was expanded in MSTO-211H spheroids, while it was barely detected in the conventional adherent culture (Fig. 1c). The ALDH^bright^ cells were also expanded in spheroids formed by MESO-4 and MESO-1. For the MESO-4 cells, a significant proportion of the cells expressed readily detectable ALDH activity under the conventional adherent conditions, and a population of cells with much higher ALDH activity appeared in the sphere-forming conditions (Supplemental Fig. S3A, S3B). In these ALDH^bright^ cells, the mRNA expressions of stem-cell-associated genes such as *SOX2*, *OCT3/4*, *NANOG*, *ZEB1*, and Z*EB2*, were upregulated compared with ALDH^dim^ cells (Fig. 1d). The expressions of other CIC-related markers, including CD133, CD24, ABCG2, and CD26, were similar in the MM cells cultured under sphere-forming versus conventional adherent conditions (Supplemental Fig. S3C). We then observed the in vivo growth of MSTO-211H cells in immunodeficient mice, and found that the MM cells cultured under spheroid conditions had higher tumorigenicity compared with those cultured under conventional adherent conditions (Fig. 1e). These results together suggested that a cell population(s) with CIC-like properties was selectively expanded in MM spheroids. We selected the MSTO-211H cells for further analysis.

### HA synthesis is upregulated in MM spheroids

**Fig. 2:**
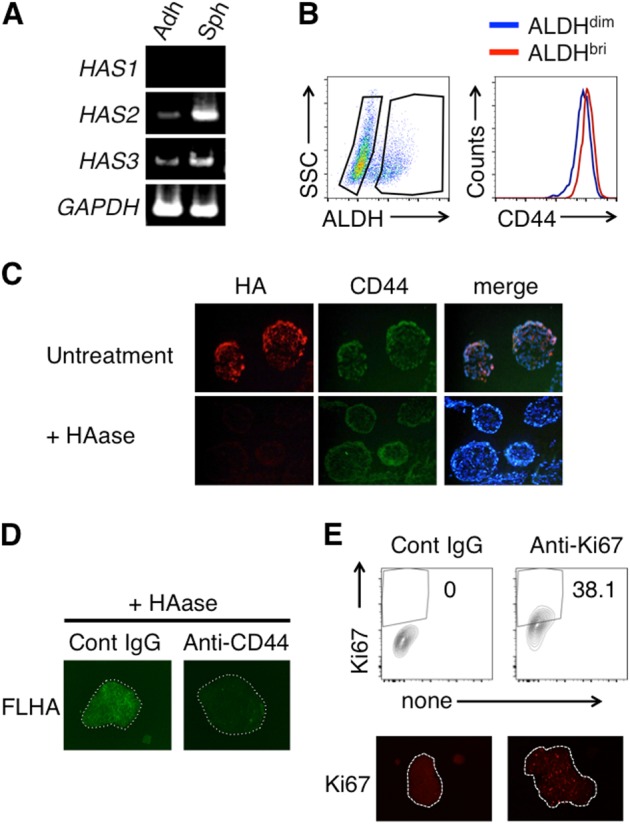
HA synthesis is upregulated in MM spheroids. **a** MSTO-211H cells were cultured for 4 days under conventional adherent or sphere-forming conditions. Total RNA was isolated from the adherent cells or spheroids, and the expression of *HAS1*, *HAS2*, and *HAS3* was determined by RT-PCR. **b** ALDH activity in MSTO-211H spheroids was determined by ALDEFLUOR assay, and the cell-surface expression of CD44 in the ALDH^bright^ (red) or ALDH^dim^ (blue) cell populations was analyzed by flow cytometry. **c** MSTO-211H spheroids were fixed with PFA and stained for hyaluronan-binding protein (HABP), anti-CD44, and DAPI (upper). To verify the specificity of the HABP staining, hyluronidase (HAase) treatment was performed before staining (lower). **d** MSTO-211H spheroids were treated with HAase and incubated with FL-HA in the presence of control or neutralizing anti-CD44. **e** Ki-67^+^ cells in MSTO-211H spheroids were analyzed by flow cytometry (upper) and immunohistochemistry (lower)

HA production has been implicated in the proliferation of MM cells [7]. To investigate the HA production by MM cells in spheroids, we examined the expression of hyaluronan synthases. As shown in Fig. 2a, the mRNA expression of *HAS2* and *HAS3* but not *HAS1* was higher in the MSTO-211H spheroids versus cells grown under adherent conditions (Fig. 2a). In the MM spheroids, the ALDH^bright^ cells and ALDH^dim^ cells expressed similar levels of *HAS2 and HAS3* (Supplemental Fig. S4). CD44, a principal receptor for HA, was also expressed on the MM cells and was slightly enhanced in the ALDH^bright^ cell population (Fig. 2b). Immunofluorescence analysis with HABP and anti-CD44 mAb showed that HA and CD44 colocalized in the MSTO-211H spheroids (Fig. 2c, upper). HAase treatment abolished the HABP binding, verifying the specificity of this analysis (Fig. 2c, lower). HAase treated spheroids bound FL-HA, which was strongly inhibited by neutralizing anti-CD44 mAb (Fig. 2d). Ki-67^+^ proliferating cells were observed in the spheroids (Fig. 2e). Together, these results suggested that the HA–CD44 interaction plays a role in the formation of MSTO-211H spheroids, which contains Ki-67^+^ proliferating cell populations.

### HA–CD44 axis contributes to the spheroid formation and maintenance of ALDH^bright^ CSCs

**Fig. 3:**
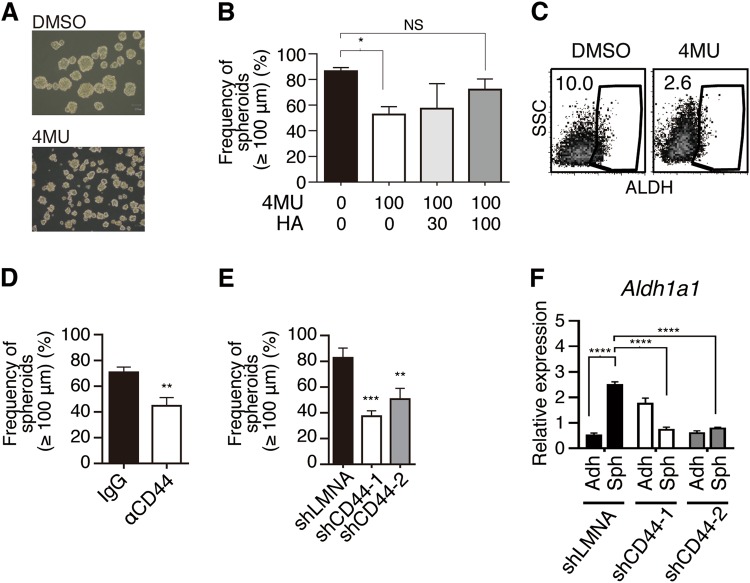
HA–CD44 axis contributes to the spheroid formation and maintenance of ALDH^bright^ CIC-like cells. **a** MSTO-211H cells were cultured for 4 days under sphere-forming conditions in the presence of the hyaluronan synthesis inhibitor, 4-methylumbelliferone (4MU, 100 μm) or DMSO (control). **b** Effects of 4MU and hyaluronan (HA) on the spheroid formation. The percentage of total spheroids (>30 μm) that were large (>100 μm in diameter) is shown. **c** MSTO-211H spheroids formed in the presence or absence of 4MU were analyzed for ALDH expression by ALDEFLUOR assay. **d** Percentage of large spheroids (>100 μm in diameter) in the presence of normal mouse IgG (control) or an anti-CD44 monoclonal antibody (10 μg/ml). **e**, **f** MSTO-211H cells were stably transfected with a plasmid for shRNA specific to CD44 (shCD44-1, shCD44-2) or to LMNA (shLMNA) as a control. **e** The shRNA-transfected MSTO-211H cells were cultured under sphere-forming conditions for 4 days, and the spheroid formation was analyzed. **f** The shRNA-transfected MSTO-211H cells were cultured in adherent (Adh) or sphere-forming (Sph) conditions for 4 days. Total RNA was isolated from each cell group, and the relative expression of *ALDH1a1* was determined by real-time PCR. Each mRNA level was normalized to that of endogenous *GAPDH*. Data are presented in triplicate as the mean ± s.d. *P < 0.05, **P < 0.01, ***P < 0.001, ****P < 0.0001

To examine whether the HA–CD44 interaction promotes the spheroid formation of MSTO-211H cells, we tested the effects of 4MU, an HA synthesis inhibitor that acts by depleting the cellular UDP-glucuronic acid, a common substrate for HASs [22]. As shown in Fig. 3a, b, 4MU inhibited the formation of large-sized spheroids. This inhibitory effect of 4MU was diminished by adding exogenous HA to the spheroid culture (Fig. 3b). Furthermore, in the presence of 4MU, the ALDH^bright^ cells under sphere-forming culture conditions decreased (Fig. 3c). These observations suggested that HA promotes both sphere formation and the expansion of the ALDH^bright^ cell population in MSTO-211H cells. To test whether CD44 plays a role in this process, we examined the effects of an anti-CD44 mAb that inhibits CD44’s binding with HA. As shown in Fig. 3d, the anti-CD44 mAb inhibited the formation of large-sized spheroids (>100 µm), as seen with 4MU. Similarly, shRNAs specific to CD44 (Supplemental Fig. S5), but not an irrelevant control shRNA (shLMNA), inhibited the formation of large-sized spheroids (Fig. 3e) and the upregulation of *ALDH* mRNA expression in MSTO-211H spheroids (Fig. 3f). An increase in *ALDH1a1* mRNA expression in serially passaged spheroids was also inhibited by shRNA for CD44 (Supplemental Fig. S6). Collectively, these results suggested that HA-mediated CD44 signaling facilitates the formation of large-sized spheroids and the expansion of the ALDH^bright^ cell population in MSTO-211H cells.

### The expressions of activin-A and its receptor subunits are upregulated in MM spheroids

**Fig. 4:**
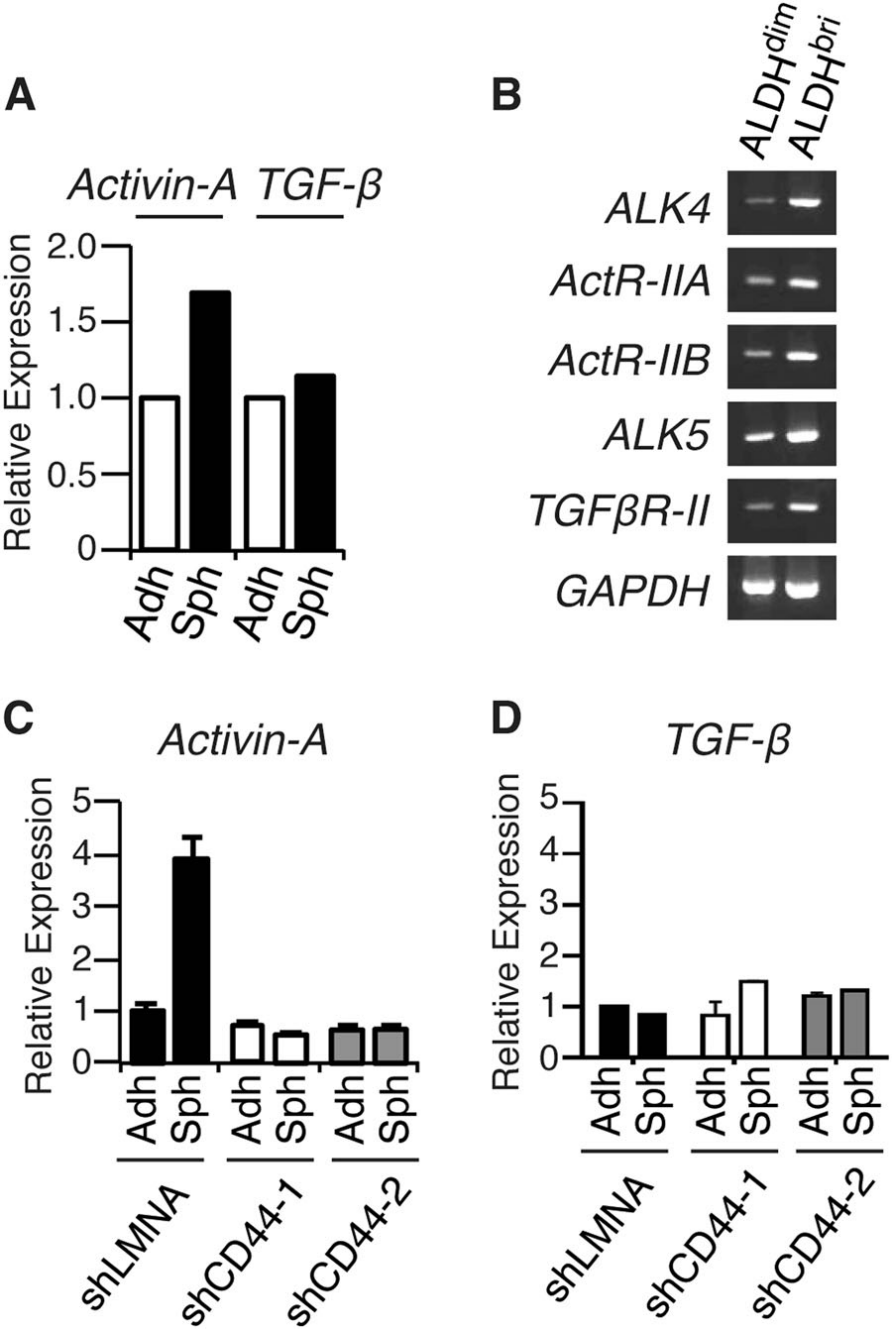
Expressions of Activin-A and its receptor subunits are upregulated in MM spheroids. **a** Total RNA was isolated from MSTO-211H adherent cells (Adh) or spheroids (Sph), and the relative expression of *ACTIVIN-A* and *TGF-β1* was determined by real-time PCR. Each mRNA level was normalized to that of endogenous *GAPDH*. **b** Total RNA was isolated from ALDH^bright^ or ALDH^dim^ cells sorted from MSTO-211H spheroids, and the expression of *ALK4*, *ActR-II, ActR-IIB*, *ALK1*, *ALK5*, *TβR-II*, and *GAPDH* was determined by RT-PCR. **c**, **d** shRNA-transfected MSTO-211H cells (shLMNA, shCD44-1, or shCD44-2) were cultured for 4 days under conventional adherent (Adh) or sphere-forming (Sph) conditions. Total RNA was isolated from each cell group, and the relative expression of *ACTIVIN-A* (**c**) and *TGF-β1* (**d**) was determined by real-time PCR. Data are presented in triplicate as the mean ± s.d.

We next investigated the regulatory factors involved in the ALDH^bright^ CIC population expansion in MSTO-211H spheroids. Members of the TGF-β cytokine family have been implicated in the maintenance and/or expansion of CIC-like cell populations in various types of cancers [23, 24]. Reverse transcriptase polymerase chain reaction (RT-PCR) analysis showed that the *ACTIVIN-A* mRNA expression was upregulated in MSTO-211H spheroids, whereas the *TGF-β* mRNA expression was unchanged (Fig. 4a). Activins are known to bind one of two type II serine threonine kinase receptors (*ActRIIA* or *ActRIIB*), which in turn recruit and phosphorylate the type I activin receptor ALK4 [25]. We therefore examined the expression of receptor subunits for activins, and found that both type II receptor subunits (ActRIIA and ActRIIB) and a type I subunit (ALK4) for activin-A were preferentially expressed in the ALDH^bright^ cells in MSTO-211H spheroids. Type II (TβR-II) and type I (ALK5/TβR-I) subunits for TGF-β were also expressed in the ALDH^bright^ cells in MSTO-211H spheroids (Fig. 4b). To examine whether signals through CD44 contributed to the up-regulation of *ACTIVIN-A* mRNA under sphere-forming conditions, we tested the effect of shRNA to CD44. As shown in Fig. 4c, d, the shRNA to CD44 abolished the upregulation of *ACTIVIN-A* mRNA expression, whereas the TGF-β mRNA was little changed. These observations suggested that the activin-A production in MSTO-211H cells is augmented by signals through CD44 under the spheroid-forming conditions, and that functional activin-A receptors are expressed in the ALDH^bright^ cell population in MSTO-211H spheroids.

### Activin-A–ALK4 axis contributes to the maintenance of ALDH^bright^ CSCs, but not to the spheroid formation of MSTO-211H cells

**Fig. 5:**
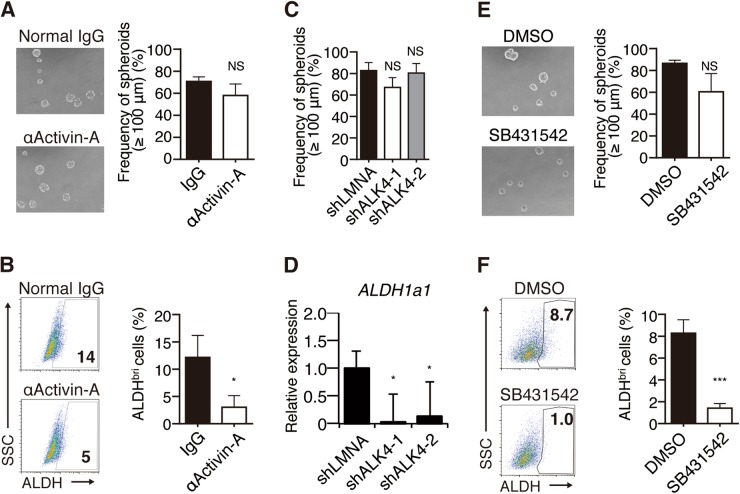
Activin-A–ALK4 axis contributes to the maintenance of CIC-like cells but not to the spheroid formation of MSTO-211H cells. **a** MSTO-211H cells were cultured for 4 days under sphere-forming conditions in the presence of normal mouse IgG (control) or anti-activin-A monoclonal antibody (10 μg/ml), and the spheroid formation was analyzed. **b** ALDH^bright^ cells in MSTO-211H spheroids formed in the presence of normal mouse IgG (control) or anti-activin-A monoclonal antibody (10 μg/ml) was determined by ALDEFLUOR assay. **c**, **d** shRNA-transfected MSTO-211H cells (shLMNA, shALK4-1, or shALK4-2) were cultured for 4 days under sphere-forming conditions. The expression of ALK4 on MSTO-211H was knocked down using shRNA (shALK4-1, shALK4-2). **c** The spheroid formation was analyzed. **d** Total RNA was isolated, and the relative expression of *ALDH1a1* was determined by real-time PCR. Each mRNA level was normalized to the endogenous *GAPDH*. **e** MSTO-211H cells were cultured for 4 days under sphere-forming conditions in the presence of DMSO (control) or ALK inhibitor, SB431542 (10 μm) and the spheroid formation was analyzed. **f** ALDH^bright^ cells in MSTO-211H spheroids in the presence of DMSO or SB431542 (10 μm) were determined by ALDEFLUOR assay. All data are presented in triplicate as the mean ± s.d. *P < 0.05, **P < 0.01, ***P < 0.001, ****P < 0.0001

We next examined the functional roles of the signals generated by activin-A and ALK4. As shown in Fig. 5a, b, a neutralizing anti-activin A mAb failed to affect spheroid formation, but it significantly decreased the ALDH^bright^ cell population. Similar effects were observed with shRNA to ALK4 (Fig. 5c, d, Supplemental Fig. S7) and with SB431542, a small-molecule inhibitor of ALK4, ALK5, and ALK7 (Fig. 5e, f). These observations indicated that signals generated by activin-A through ALK4 are important for the expansion of ALDH^bright^ CICs but not for the spheroid formation of MSTO-211H cells.

### Signals through CD44 and ALK4 play critical roles in tumor growth in vivo

**Fig. 6:**
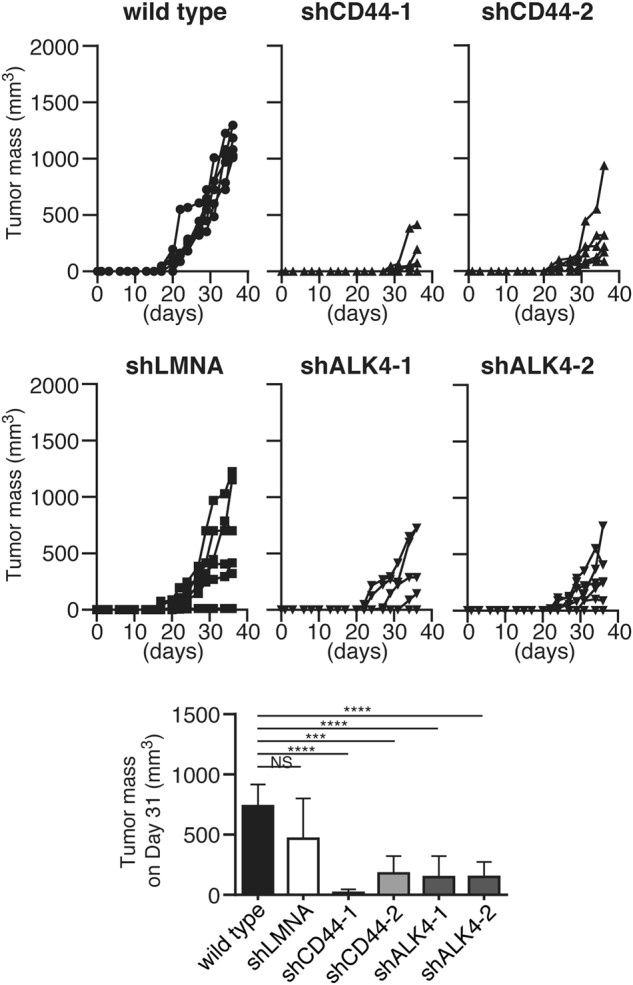
Signals through CD44 and ALK4 are required for optimal tumor growth in vivo. **a**, **b** Anti-CD122 (TM-β1: 500 μg/body) was intraperitoneally injected into SCID mice to deplete NK cells before implantation. Seven days later, MSTO-211H cells (parental [wild type] or shRNA-transfected cells [shCD44-1, shCD44-2, shALK4-1, shALK4-2, and shLMNA as a negative control]) (5 × 10^4^ cells) were subcutaneously injected into the mice, and the tumors were measured every 3–4 days (*n* = 6). Tumor mass was calculated as described in Materials and methods. **a** Tumor mass of individual mice. **b** Mean value of tumor mass in each group (day 31). *P < 0.05, **P < 0.01, ***P < 0.001, ****P < 0.0001

We next investigated the significance of the signals mediated by CD44 or ALK4 for the tumorigenicity of MSTO-211H cells in immunodeficient mice. As shown in Fig. 6a, b, shRNAs for CD44 and ALK4 substantially inhibited the in vivo growth of MSTO-211H cells. The shRNA to LMNA used as an irrelevant control appeared to exert some inhibitory effect on the in vivo growth of MSTO-211H cells in immunodeficient mice, but it did not reach significance. These observations together indicated that signals through CD44 and ALK4 were interrelated and both pathways play critical roles in growth of MSTO-211H cells in vivo.

## Discussion

In the present study, we showed that ALDH^bright^ CICs expressing stem-cell-associated genes, including *SOX2, OCT3/4, NANOG*, *ZEB1*, and *ZEB2*, were enriched and expanded in spheroids formed by MM cells (MSTO-211H). The expression of HA synthases *HAS2* and *HAS3* was upregulated in the MM spheroids, and HA-mediated signals through CD44 were important for the formation of large-sized spheroids and the expansion of ALDH^bright^ CICs. The expression of *ACTIVIN-A*, a TGF-β cytokine member, was also upregulated in the spheroids, and signals through the type I activin-A receptor subunit (ALK4) contributed to the maintenance of ALDH^bright^ CICs, but not to spheroid formation. These results together suggested that signaling pathways through CD44 and ALK4 differentially regulate the spheroid formation and ALDH^bright^ CICs in MM cells, and that possible cooperation exists where HA–CD44 axis may locate upstream of the Activin-A–Alk4 axis in tumor formation (Supplemental Fig. S8). We also found that CD44- and ALK4-mediated signals are both required for optimal tumor growth in immunodeficient hosts.

High ALDH activity is widely used to characterize subpopulations of cancer cells with CIC-like properties [26]. We and others [19, 20] have used high ALDH activity and/or *ALDH1* mRNA expression to characterize the MM cell population with CIC-like properties. Cortes-Dericks et al. [19] and Shapiro et al. [20] identified cell populations with CIC-like properties in various MM cell lines that had similar properties, such as high sphere-forming activity and tumorigenic potential, to those of the ALDH^bright^ CICs in the MM cells in this study. However, the regulatory mechanisms underlying the maintenance and expansion of the CIC population in MM remained to be fully elucidated.

The interaction of HA with its principal receptor CD44 has been implicated in the progression of a wide variety of tumors [27, 28]. In MM patients, a high concentration of HA in the pleural effusion fluid or serum and an overexpression of CD44 were reported [7]. In addition, MM spheroids observed in the pleural effusion fluid have been shown to associate with increased malignancy [29, 30]. The interaction of HA with CD44 enhances the cell proliferation and invasive properties of MM cells, and may contribute to the disease progression [31]. We showed in this study that MM cells in spheroids exhibited an increased expression of the HA synthases *HAS2* and *HAS3* and autonomously produced HA, and that ALDH^bright^ CICs in the spheroids expressed high levels of CD44. Importantly, inhibiting either the HA synthesis or CD44 function by shRNA-mediated knockdown or a neutralizing antibody abolished the formation of large-sized MM spheroids, the upregulation of activin-A in the spheroid-forming cells, and the expansion of ALDH^bright^ CICs. Our observations thus suggest that MM cells in the spheroids acquire the capability to autonomously synthesize HA and produce the HA-rich microenvironment that supports the expansion of ALDH^bright^ CICs, although detailed molecular organization of HA-rich pericellular matrix and direct interaction of CD44 and HA in situ remained to be determined. The signals that upregulate the HAS2 and HAS3 expression in MM spheroids are presently unknown. In this regard, it is noteworthy that a recent study by Liu et al. [32] showed that CD44-mediated signaling generates a positive feedback loop by inducing HAS2 expression through activation of the PI3K/AKT pathway. Because CD44 is required for EMT and CD44 signals activate systems that facilitate cell survival[33], further investigations are required to elucidate the significance of the HA–CD44 interaction in the progression and invasion of MM.

Members of the TGF-β cytokine family, including bone morphogenic proteins, TGF-β, nodal, and activin, exert multiple effects on tumor cells, depending on the cell type and cellular context [23, 34]. Previous studies showed that deregulated activin A signals contribute to a malignant phenotype of MM [9, 10] and that the plasma activin-A level is a prognostic marker for the disease [11]. We showed in this study that the expression of activin-A was upregulated in MM cells under sphere-forming conditions. Notably, we found that the type I activin-A receptor ALK4 was selectively upregulated in the ALDH^bright^ CICs in MM cells, and that the neutralization of activin-A and functional inactivation of ALK4 by shRNA or a low-molecular-weight inhibitor diminished the ALDH^bright^ CIC population without affecting the spheroid formation of MM cells. Thus, our observations confirmed the role of activin-A signals in MM and extended their significance in a CIC population of MM. Although the biological importance of signals provided by other TGF-β family members remains to be experimentally tested, the mechanisms of activin-A-mediated and ALK4-driven signaling pathways in maintaining and expanding the CIC compartment of MM cells particularly warrant further investigation.

We showed in this study that MM spheroids contained highly tumorigenic cell populations. The knockdown of CD44 or ALK4 with specific shRNAs largely eliminated the tumor growth in immunodeficient xenogenic hosts, indicated that signals through CD44 and ALK4 are interrelated and critical for tumor growth in vivo. Recent studies reported that CD44 is a direct transcriptional target of YAP-TEAD complex that is negatively regulated by the Hippo pathway that is frequently inactivated in MM cells including the MSTO-211H cells used in this study [35, 36]. Our observations further support the notion that CD44 has a critical role in the growth of MM cells in vivo, probably through its interaction with HA produced by the MM cells.

Previous studies demonstrated that activin-A regulates the migration and growth of MM cells [9, 10]. We showed in this study that the type I receptor ALK4 was selectively expressed in ALDH^bright^ CICs along with the type II receptor ActRIIB and ActRIIB and that the shRNA-mediated knockdown of ALK4 significantly inhibited tumor growth in immunodeficient mice. Our results thus suggest that ALK4 signals contribute to the in vivo tumorigenicity of MM cells through their activation of ALDH^bright^ CICs. Signals through ALK4 have also been implicated in the self-renewal of CICs in colorectal and pancreatic cancers [23, 24].

In summary, we showed in this study that the HA–CD44 pathway and activin-A–ALK4 pathway differentially contribute to the autonomous formation of the protumorigenic microenvironment and the expansion/maintenance of the CIC population in MM cells, respectively. Both signaling pathways are critical for the in vivo tumorigenesis of the MM cells. Our findings suggest that therapeutic approaches targeting the HA–CD44 and activin-A–ALK-4 pathways could be effective strategies for suppressing the CIC compartment in MM.

## Materials and methods

### Human MM cell lines

MSTO-211H (biphasic), NCI-H28 (epithelioid), and NCI-H2052 (sarcomatoid) were purchased from the ATCC. ACC-MESO-1 (sarcomatoid) and ACC-MESO-4 (epithelioid) were obtained from RIKEN Cell Bank. All cells were cultured in RPMI 1640 medium (Sigma) supplemented with 2 mM l-glutamine (Gibco), 100 units/ml penicillin, 100 µg/ml streptomycin (Gibco), 10 mM 4-(2-hydroxyethyl)-1-piperazineethanesulfonic acid (HEPES) (Gibco), 1 mM sodium pyruvate (Gibco), minimum essential medium/nonessential amino acids (Gibco), and 10% heat-inactivated FBS (Gibco), in humidified CO_2_ incubators at 37 °C. All cells used in this study were use in the experiments within 4 weeks of culture, and were tested for mycoplasma contamination using the DAPI staining method.

### Sphere formation of MM cells

The cells were washed with phosphate-buffered saline (PBS), resuspended (1.0 × 10^4^ cells/ml) in DMEM/F-12 (Gibco) supplemented with 50 ng/ml EGF (R&D), 10 ng/ml bFGF (R&D), 25 μg/ml insulin (Gibco), and 2% B27 supplement (Gibco), and cultured for 4 days in ultra-low attachment surface plates (Corning) [21]. Images of spheroids were acquired with a BZ-700×0 microscope (Keyence), and the Heywood diameter of the spheroids was determined. The number of spheroids (>30 or >100 μm in Heywood diameter) in 5 low-power fields (×100) was counted [35, 37]. Frequency of spheroids (>100 μm) (%) was calculated as follows: [the number of spheroid (>100 μm)/the total number of spheroids (>30 μm)] × 100. In some experiments, 4-methylumbelliferone (4MU) (100 μΜ) (Sigma), hyaluronan (100 μg/ml) (Seikagaku Corp.), SB431542 (10 μm) (R&D), anti-CD44 monoclonal antibody (mAb) (clone: BRIC235, 10 μg/ml) (IBGRL), or anti-activin A (betaA subunit) mAb (Clone: #69403, 10 μg/ml) (R&D) was added to the culture medium.

### Flow cytometry

For the analyses of cell-surface CD44 expression, cells (5.0 × 10^5^) were stained with a PE-conjugated anti-human CD44 mAb (clone: IM7; BioLegend) for 60 min on ice. After washing the cells with PBS, the fluorescence intensity of individual cells was determined using a FACSAria II flow cytometer (Becton Dickinson). FACSDiva software (Becton Dickinson) and Flowjo software (Treestar) were used for data acquisition and analysis, respectively. ALDH activity was assayed using the ALDEFLUOR kit (Stem Cell Technologies), according to the manufacturer’s instructions. Briefly, cells (1.0 × 10^6^) were incubated in assay buffer containing the ALDH substrate BODIPY-amino acetaldehyde (BAAA), and an aliquot of the ALDH substrate-treated cells was immediately quenched with a specific ALDH inhibitor, diethylaminobenzaldehyde (DEAB), and used as a negative control. After a 45-min incubation at 37 °C, the ALDH^bright^ or ALDH^dim^ cells were analyzed or sorted with the FACSAria II flow cytometer. For the analyses of intracellular Ki-67 expression, spheroids were dispersed with spheroid dispersion solution (SCIVAX) at 37 °C for 15 min to obtain a single-cell suspension. Subsequently, cells were fixed with cold 4% PFA in PBS. After blocking with Block Ace (DS Pharma Biomedical) and permeabilization with 0.1% saponin in PBS, the spheroid-derived single cells were stained with anti-Ki-67 mAb (Becton Dickinson) and PE-conjugated anti-mouse IgG (Jackson Immunoresearch). After washing the cells with PBS, the fluorescence intensity of individual cells was measured with a FACSAria II flow cytometer.

### PCR analysis

The total RNA was isolated from cells using an RNeasy Plus Mini Kit (Qiagen), and cDNA was obtained from 500 ng of the total RNA using the PrimeScript II 1st strand cDNA Synthesis Kit (TaKaRa). Real-time quantitative RT-PCR was performed with a PRISM 7700 (Applied Biosystems) using the Power SYBR Green PCR Master Mix (Applied Biosystems). The expression of mRNA was normalized to the expression of GAPDH mRNA. The primer sequences for human genes were as follows: *gapdh*, 5′-gaccccttcattgacctcaac-3′ and 5′-cttctccatggtggtgaaga-3′; *sox2*, 5′-ggaaatgggaggggtgcaaaagagg-3′ and 5′-ttgcgtgagtgtggatgggattggtg-3′; *nanog*, 5′-aatacctcagcctccagcagatg-3′ and 5′-tgcgtcacaccattgctattcttc-3′; *oct3/4*, 5′-gacagggggaggggaggagctagg-3′ and 5′-cttccctccaaccagttgccccaaac-3′; *zeb-1*, 5′-tttggctggatcactttcaag-3′ and 5′-gccaataagcaaacgattctg-3′; *zeb-2*, 5′-ggtccagatcgaagcagctcaat-3′ and 5′-gtgacttctatgtttgttcacatt-3′; *has1*, 5′-ccactgtgtatcctgcatca-3′ and 5′-cttggtagcataacccatgc-3′; *has2*, 5′-atggggtggaaaaagagaag-3′ and 5′-tgaggaatgagatccaggaa-3′; *has3*, 5′-ttggctgtgtgcagtgtattagt-3′ and 5′-ggtctctgtgaggcacttgg-3′; *aldh1a1*, 5′-ctcgattggatggcagtagct-3′ and 5′-aacactgtgggctggacaaa-3′; *activin-a*, 5′-aaagcttcatgtgggcaaag-3′ and 5′-aatctcgaagtgcagcgtct-3′; *tgf-β1*, 5′-ggctttcgccttagcgccca-3′ and 5′-ctcggcggccggtagtgaac-3′; *alk-4*, 5′-ggagcgtcttgtctttggag-3′ and 5′-tgcaacaggatcgacttgag-3′; *alk-5*, 5′-gatgggctctgctttgtctc-3′ and 5′-caaggccaggtgatgacttt-3′; *actR-IIA*, 5′-gcgagaacttcctccggatt-3′ and 5′-atagcacctgaagaacaggaga-3′; *actR-IIB*, 5′-gtctattgcccacagggact-3′ and 5′-ctcaggagccatgtaccgtc-3′; *TgfβR-II*, 5′-ctcaggagccatgtaccgtc-3′ and 5′-ctcggcggccggtagtgaac-3′.

### Immunostaining

Spheroids were fixed with cold 4% PFA in PBS. After blocking with Block Ace (DS Pharma Biomedical), the spheroids were permeabilized with 0.1% saponin and stained with FITC-conjugated anti-CD44 mAb (clone; IM7, BioLegend) plus biotin-conjugated hyaluronan-binding protein (HABP) (Seikagaku Corp.) After a PBS wash, the spheroids were stained with Alexa594-conjugated streptavidin and 4′, 6-diamidino-2-phenylindole (DAPI). For the analyses of Ki-67 expression, anti-Ki-67 mAb (Becton Dickinson) and Alexa594-conjugated anti-mouse IgG (Molecular Probes) were used. In some experiments, spheroids were treated with hyaluronidase (100 U/ml; Sigma) and incubated with FITC-labeled HA (FL-HA; Seikagaku Corp.) in the presence or absence of anti-CD44 mAb (clone; BRIC 235). Fluorescent images were taken with the BZ-7000 microscope (Keyence). The specificity of the HABP was confirmed by hyaluronidase treatment before HABP staining.

### Plasmid construction and shRNA-mediated knockdown

The shRNA-target sequences against human CD44, human ALK4, and lamin A/C (LMNA), were designed by an online tool, the BLOCK-iT RNAi Designer (Invitrogen). The pre-designed oligonucleotides (Hmi402590 and Hmi402591 for human CD44 and Hmi400217 and Hmi400219 for human ALK4) were annealed and cloned into the pcDNA6.2-Gw/EmGFP-miR expression vector (Invitrogen). The resultant plasmid for knockdown was transfected into MSTO-211H cells using Lipofectamine 2000 (Invitrogen), according to the manufacturer’s instructions. After transfection, GFP-positive cells were sorted by the FACSAria II cell sorter.

### In vivo tumor growth

The mice were randomly distributed into each group before treatment. NOD. CB17-*Prkdc*^scid^/J (NOD/SCID) mice and CB17/lcr-*Prkdc*^scid^/CrlCrlj (SCID) mice (Charles River) were pre-treated with an intraperitoneal injection of anti-CD122 mAb (clone; TM-β1, 500 μg/body, prepared in our laboratory) to deplete NK cells seven days before tumor inoculation [38]. For a comparison of the in vivo growth of MSTO-211H cells cultured under adherent or sphere-forming conditions, the cells were collected and washed in PBS, and equal number of cells (1 × 10^3^ cells) in 100 μl of PBS in the form of single-cell suspension (from adherent condition) or spheroid (from sphere-forming condition) were injected into the NK-depleted NOD/SCID mice using a 23-guage needle. Numbers of MSTO-211H cell in the form of spheroids were pre-determined in a separate experiment. To evaluate effects of gene knockdown in MSTO-211H cells, wild type and the MSTO-211H cells transfected with shRNA constructs cultured under adherent conditions were collected and washed in PBS, and the cells (5 × 10^4^ cells) in 100 μl of PBS in the form of single-cell suspension were injected into the NK-depleted SCID mice. Tumor mass was calculated by the formula: tumor mass (mm^3^) = length (mm) × width (mm)^2^ × 1/2. For animal studies, no blinding was done. All animal experiments were approved by the institutional animal care and use committee at Hyogo University of Health Sciences (approval numbers 2008-03, 2010-09, 2013-02, 2013-22).

### Statistical analysis

For each experimental technique, unless otherwise stated in the Materials and methods or figure legends, individual experiments were repeated three or more times; this allowed us to reach high statistical significance in all the reported assays. No statistical methods were used for sample size selection. The variances were similar between the groups under comparison in all these cases. No samples or animals were excluded from the analysis. Statistical analyses were performed using GraphPad Prism version 6.0 software (GraphPad). Two-tailed Student’s *t*-test was used to compare two groups. One- or two-way ANOVA was performed to compare the values of >2 groups. The statistical significance was set at *P* < 0.05.

## Electronic supplementary material

supplemental figures
