# Supplementary material for: Differential regulation of the sphere formation and maintenance of cancer-initiating cells of malignant mesothelioma via CD44 and ALK4 signaling pathways
Source: Oncogene. 2018 Jul 30;37(49):6357–67. doi: 10.1038/s41388-018-0405-y (PMC6283855; doi:10.1038/s41388-018-0405-y)
Supplement: Supplementary file 1 — supplemental figures [file 41388_2018_405_MOESM1_ESM.pdf]

Figure S1

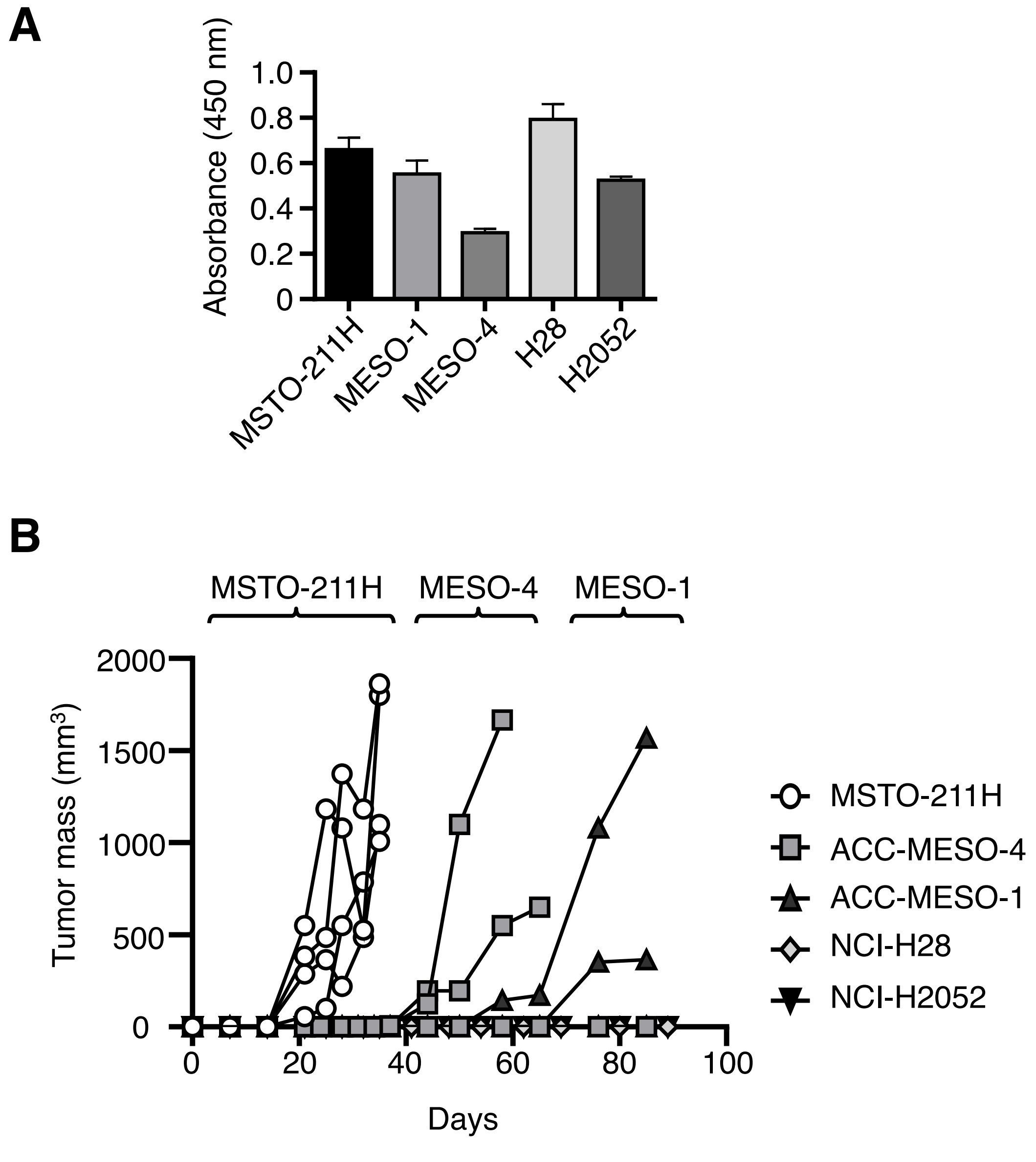

Supplemental Figure S1

(A) MM cells (MSTO-211H, ACC-MESO-1, ACC-MESO-4, NCI-H28, and NCI-H2052) were plated in 96-well plates at 1.0 x 10^3 cells per well and cultured. After 3 days, the viable cells were stained using MTT reagent, and the absorbance at 450 nm was measured using a microplate reader. (B) Anti-CD122 (TM-β1; 500 μg/body) was intraperitoneally injected into NOD/SCID mice to deplete NK cells before tumor implantation. Seven days later, MSTO-211H, ACC-MESO-1, ACC-MESO-4, NCI-H28, or NCI-H2052 cells (3.0 x 10^6 cells) were subcutaneously injected into the mice. The tumor growth *in vivo* was measured every 3-4 days (n=4). Data are presented as the mean ± s.d.

Figure S2

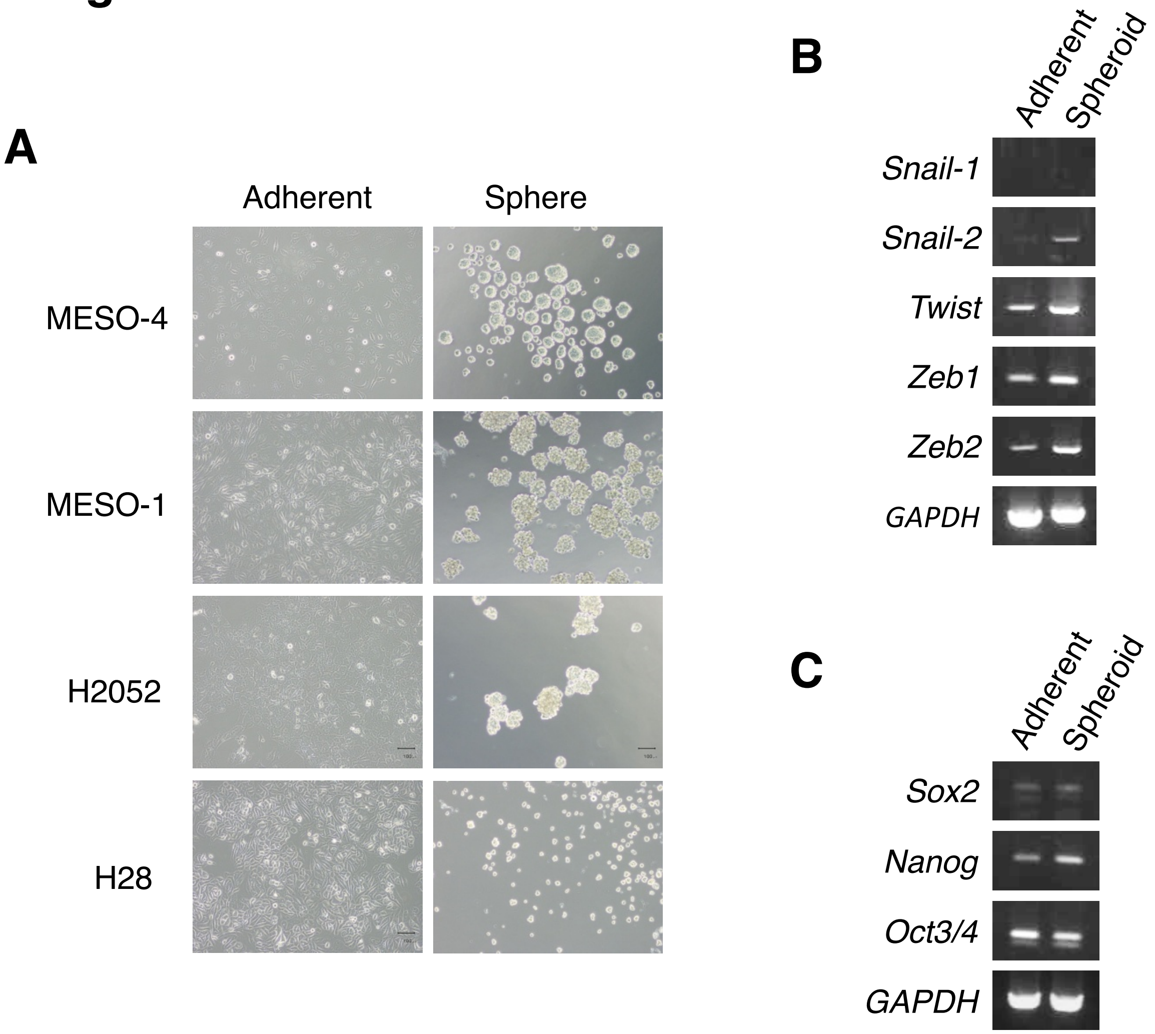

Supplemental Figure S2

(A) ACC-MESO-4, ACC-MESO-1, NCI-H2052, and NCI-H28 cells were cultured for 4 days under conventional adherent (left) or sphere-forming conditions (right). (B, C) Total RNA was isolated from adherent or spheroid-forming MESO-4 cells. The expression of EMT- (*SNAIL-1*, *SNAIL-2*, *TWIST, ZEB-1, and ZEB-2*) and stemness-associated genes (*SOX2*, *NANOG*, *OCT3/4*) was determined by RT-PCR. Data shown are representative of at least three independent experiments.

Figure S3

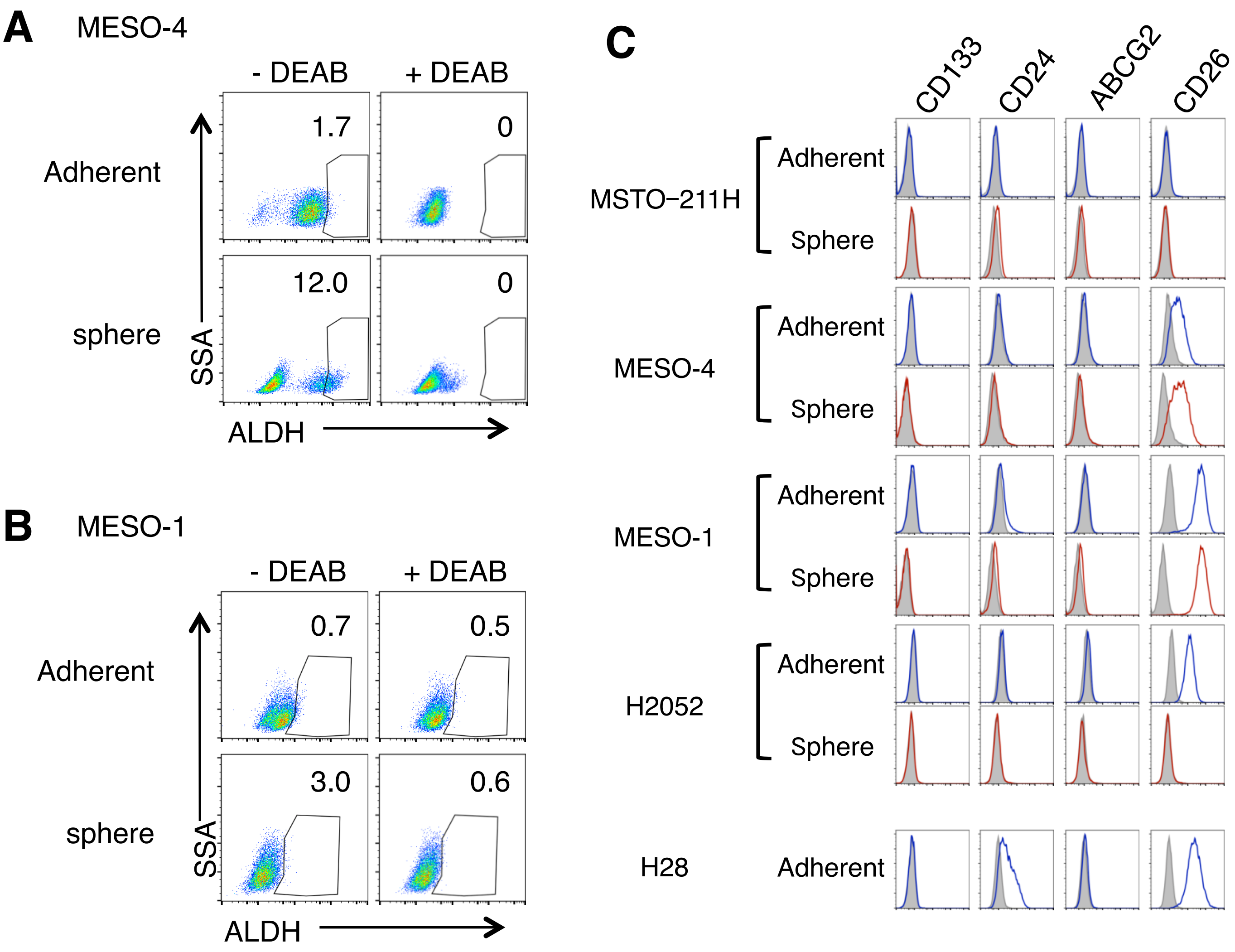

Supplemental Figure S3

(A, B) ALDH activity of ACC-MESO-4 (A) and ACC-MESO-1 (B) adherent cells and spheroids was determined by ALDEFLUOR assay. In MESO-4, ALDH^{bright} gated cells were indicated as cells with a high level of ALDH expression. In MESO-1, cell populations that disappeared with a specific ALDH inhibitor, diethylaminobenzaldehyde (DEAB) were gated as ALDH^{bright} cells. (C) Cell-surface expression of CIC-related markers (CD133, CD24, ABCG2, and CD26) on MM cells (MSTO-211H, MESO-1, MESO-4, H2052, and H28) cultured for 4 days under the adherent or sphere conditions was analyzed by flow cytometry. Unstained control (gray filled) and specific mAb staining (blue or red open) histograms are shown. Data shown are representative of at least three independent experiments.

Figure S4

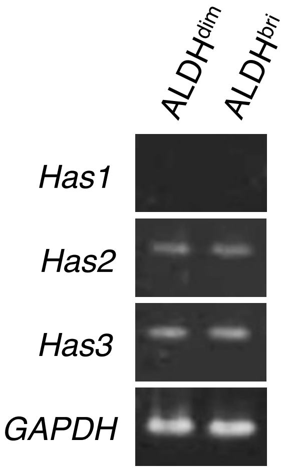

Supplemental Figure S4
Total RNA was isolated from the ALDH^{dim} or ALDH^{bright} cell populations in MSTO-211 spheroids, and the expression of *HAS1*, *HAS2*, and *HAS3* was determined by RT-PCR. Data shown are representative of at least three independent experiments.

Figure S5

A

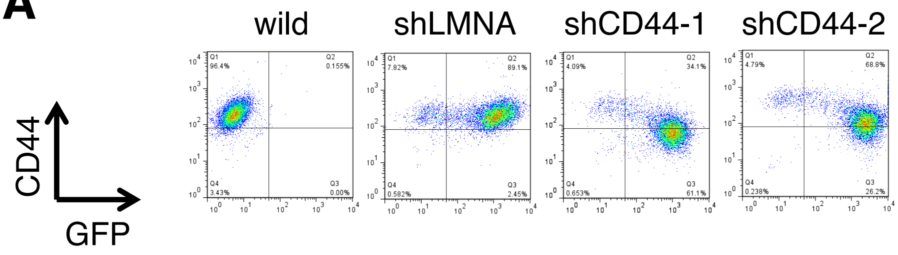

B

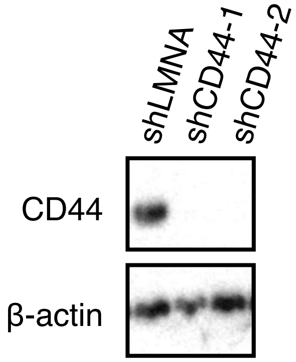

Supplemental Figure S5

(A) MSTO-211H cells were stably transfected with a plasmid for shRNA specific to CD44 (shCD44-1, shCD44-2) or LMNA (shLMNA) as a control. The cell-surface expression of CD44 on shRNA-transfected cells (GFP^+) was analyzed by flow cytometry. (B) Total CD44 protein expression of shRNA-transfected cells was analyzed by western blotting. β-actin was detected as an endogenous control.

Figure S6

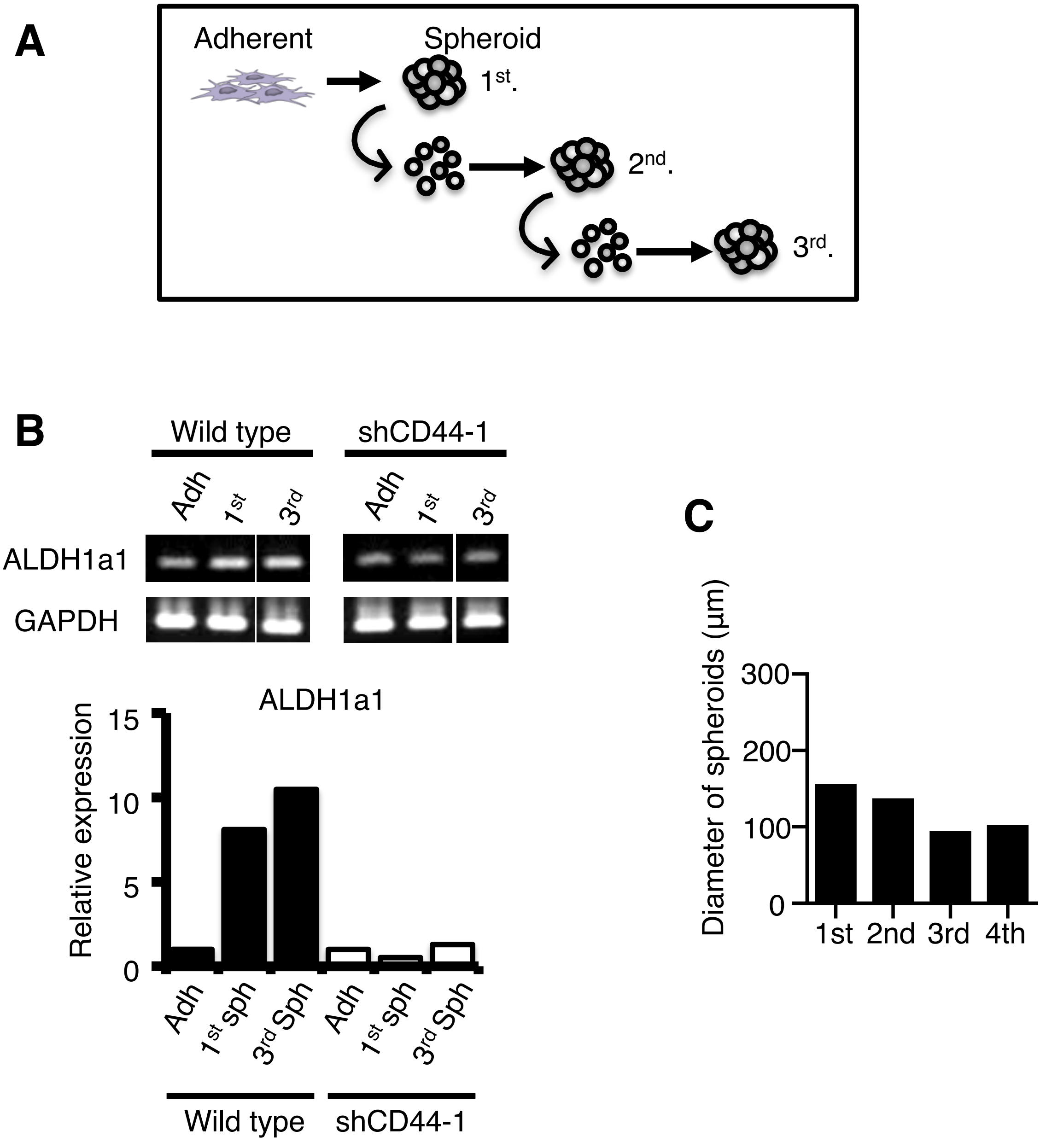

Supplemental Figure S6

(A) Serial passage of spheroid cells. Control or CD44 shRNA-transfected MSTO-211H cells were cultured for 4 days under sphere-forming conditions. For secondary or tertiary spheroid formation, spheroids from control or CD44 shRNA-transfected MSTO-211H cells were dispersed with spheroid dispersion solution (SCIVAX). The single cell suspensions obtained from original spheroids were then re-cultured for 4 days under sphere-forming conditions, again. (B) Total RNA was isolated from adherent cells or spheroids (1st and 3rd passage), and the relative expression of *ALDH1a1* was determined by real time PCR. Each mRNA level was normalized to that of endogenous *GAPDH*. (C) The diameter of serially passaged spheroid was measured. Images of spheroids (5 low-power fields [x100]) were acquired and the mean Heywood diameter of the spheroids was determined.

Figure S7

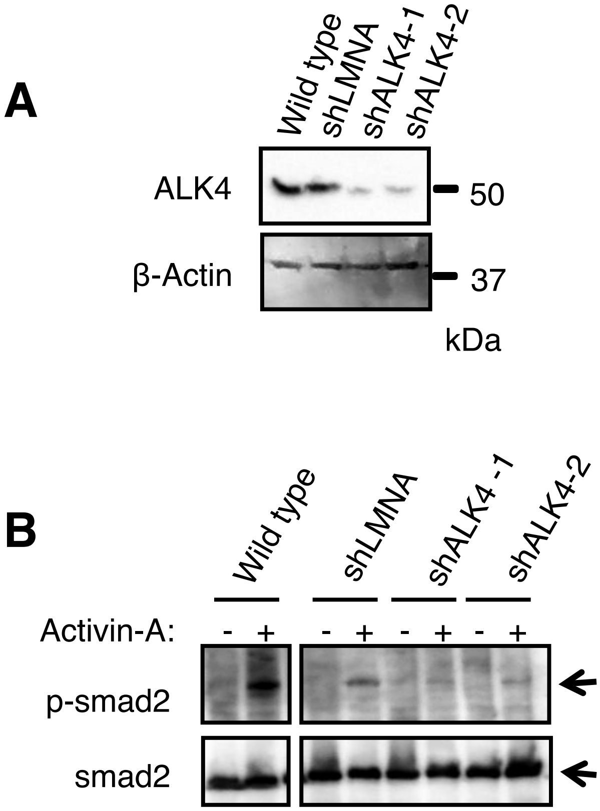

Supplemental Figure S7

(A) MSTO-211H cells were stably transfected with a plasmid for shRNAs specific to ALK4 (shALK4-1, shALK4-2) or shLMNA as a control, and the total ALK4 protein expression in the shRNA-transfected cells was analyzed by western blotting. β-actin was detected as an endogenous control. (B) shRNA-transfected MSTO-211H cells were cultured in serum-free medium for 60 min, and then stimulated with activin-A (10 ng/ml) for 30 min. Cells were collected and the total cell lysate was extracted with RIPA. Phospho-smad2 and total smad2 were analyzed by western blotting.

Figure S8

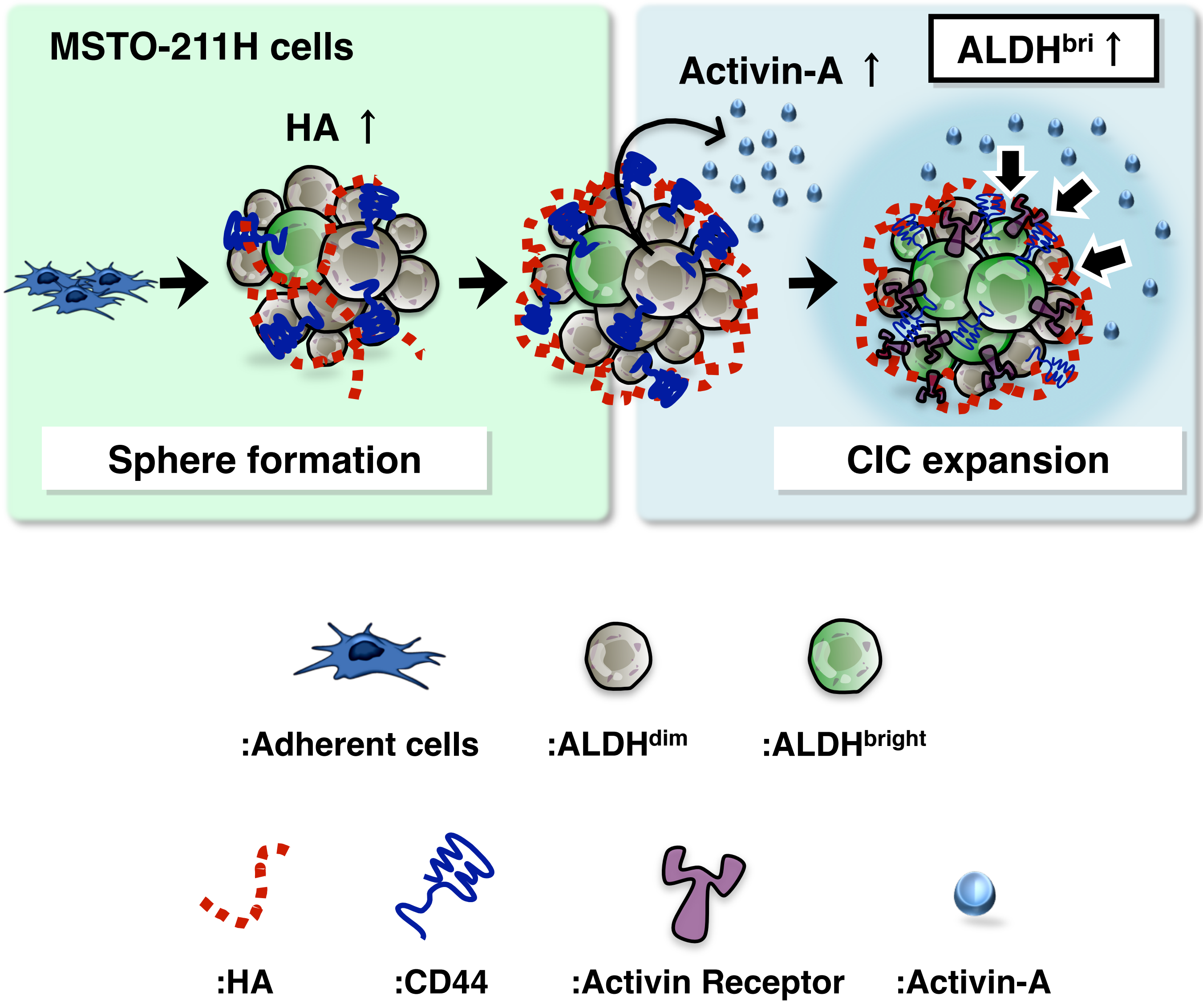

Supplemental Figure S8
A graphical summary of regulatory mechanisms of sphere formation and maintenance of CICs in malignant mesothelioma (MSTO-211H) cells. In this model, HA-CD44 and Activin-A-ALK4 pathways differentially regulate the spheroid formation and maintenance of $ALDH^{bright}$ CICs.
